# Pituitary adenylate cyclase-activating polypeptide and mitochondrial homeostasis in neuronal injury: mechanisms and translational prospects

**DOI:** 10.3389/fimmu.2026.1893747

**Published:** 2026-08-03

**Authors:** Wuping Sun, Ruyue Mo, Mingzhu Zhai

**Affiliations:** 1 Department of Clinical Laboratory Medicine, Fifth Affiliated Hospital of Southern Medical University, Guangzhou, China; 2 Center for Medical Experiments (CME), Shenzhen University of Advanced Technology General Hospital, Shenzhen, China

**Keywords:** cerebral ischemia, mitochondrial dynamics, mitochondrial dysfunction, neuroprotection, oxidative stress, PACAP, pituitary adenylate cyclase-activating polypeptide, retinal neuropathy

## Abstract

Pituitary adenylate cyclase-activating peptide (PACAP) is a pleiotropic neuropeptide widely distributed in the nervous system, exhibiting potent cytoprotective effects across a spectrum of neurological disorders. Its neuroprotection is largely mediated through three G protein-coupled receptors (PAC1, VPAC1, VPAC2), activating downstream pathways that converge on preserving mitochondrial integrity. Mitochondrial dysfunction, characterized by bioenergetic failure, oxidative stress, perturbed dynamics (such as fusion and fission), and impaired quality control, is a hallmark of traumatic nerve injury, cerebral ischemia, and retinal neuropathy. This review systematically synthesizes recent evidence elucidating how PACAP counteracts these pathological processes. We detail its mechanisms in 1) mitigating neuropathic pain and promoting axonal regeneration after peripheral nerve trauma; 2) attenuating excitotoxicity, apoptosis, and neuroinflammation following cerebral ischemia by regulating mitochondrial permeability, fission/fusion balance, and NLRP3 inflammasome activation; and 3) protecting retinal ganglion cells against diabetic retinopathy and glaucomatous damage via modulating oxidative stress and apoptotic signaling. Furthermore, we discuss the translational potential of PACAP, including its biomarker value in cerebrospinal fluid and plasma for injury prognosis, and the promise of innovative delivery routes to enhance brain bioavailability. By focusing on mitochondrial-centric mechanisms, this review underscores PACAP as a neuroprotective regulator and highlights its candidacy for developing next-generation neurotherapeutics.

## Introduction

Mitochondria are indispensable for neuronal survival and function. In addition to generating adenosine triphosphate (ATP), mitochondria regulate calcium homeostasis, reactive oxygen species (ROS) signaling, apoptotic cascades, inflammatory responses, and local metabolic support for axons and synapses. Neurons are particularly dependent on mitochondrial integrity because of their high energy demand, polarized morphology, long axonal projections, and limited regenerative capacity. Mitochondrial transport, fusion, fission, and turnover must therefore be precisely coordinated to maintain synaptic activity, axonal maintenance, and cellular adaptation to injury (1).

Mitochondrial dysfunction is now recognized as a convergent pathological mechanism in a broad spectrum of neurological disorders, including traumatic nerve injury, cerebral ischemia, retinal neuropathy, neurodegenerative disease, and inflammatory nervous system injury (2). In these conditions, mitochondria shift from maintaining neuronal homeostasis to amplifying cell injury. Impaired oxidative phosphorylation leads to ATP depletion; calcium overload and oxidative stress trigger mitochondrial permeability transition pore (MPTP) opening; excessive mitochondrial fission fragments the mitochondrial network; defective mitophagy prevents removal of damaged mitochondria; and release of cytochrome c or mitochondrial DNA activates apoptotic and inflammatory cascades. Thus, mitochondria represent not only a marker of neuronal injury but also a therapeutic hub for neuroprotection.

Pituitary adenylate cyclase-activating polypeptide (PACAP) is a biologically active neuropeptide first identified in the hypothalamus by Arimura and colleagues (3). It is encoded by the Adcyap1 gene and exists mainly in two mature forms, PACAP-38 and PACAP-27, with PACAP-38 being the predominant form in mammalian tissues (4, 5). PACAP exerts its biological effects through three G protein-coupled receptors: PACAP receptor type 1 (PAC1) and vasoactive intestinal peptide receptors 1 and 2 (VPAC1 and VPAC2). Among these receptors, PAC1 has the highest affinity for PACAP and is strongly associated with neuroprotective signaling (6). PACAP is widely expressed in the central and peripheral nervous systems and participates in neuronal proliferation, differentiation, migration, survival, synaptic plasticity, and injury repair (6, 7). A growing body of evidence has demonstrated the protective effects of PACAP in cerebral ischemia, intracerebral hemorrhage, subarachnoid hemorrhage, retinal injury, traumatic brain injury, peripheral nerve injury, and neuropathic pain (8–13). Recent studies have increasingly emphasized the relationship between PACAP and mitochondrial homeostasis, suggesting that PACAP-mediated neuroprotection is closely linked to preservation of mitochondrial function (3). However, many previous reviews have addressed PACAP in a largely descriptive manner, without fully integrating common mitochondrial cell death mechanisms, disease-specific examples, and translational barriers.

Accordingly, this review is organized around a stepwise mechanistic-to-translational framework. We first define the shared mitochondrial dysfunction-driven cell death cascades that link traumatic nerve injury, cerebral ischemia, and retinal neuropathy. We then discuss how PACAP signaling may counter these convergent mitochondrial defects by coordinating bioenergetic recovery, redox balance, mitochondrial dynamics, mitophagy, apoptosis, and inflammatory control. Building on this mechanistic foundation, we summarize key preclinical evidence across disease models in a comparative table. Finally, we analyze the main translational barriers and propose future therapeutic strategies needed to advance PACAP toward clinically viable neurotherapeutics.

## Methods for literature retrieval

A comprehensive literature search was conducted in PubMed, Web of Science Core Collection, Embase, and China National Knowledge Infrastructure (CNKI), covering publications from January 2010 to June 2026. The search combined “pituitary adenylate cyclase-activating polypeptide” or “PACAP” with terms related to mitochondrial dysfunction (“mitochondria”, “mitophagy”, and “mitochondrial dynamics”) and neurological injury (“neuroprotection”, “traumatic nerve injury”, “cerebral ischemia”, “stroke”, “retinal neuropathy”, “glaucoma”, and “diabetic retinopathy”). Original preclinical studies, translational or clinical biomarker studies, and mechanistic or systematic reviews relevant to PACAP-mediated mitochondrial protection were included. Conference abstracts, case reports, duplicate publications, studies focused exclusively on non-neural peripheral PACAP functions, and articles lacking mitochondrial pathway-related data were excluded.

### Mitochondrial dysfunction-driven cell death cascades in neurological injury

Mitochondria are central integrators of neuronal survival and death signaling. In neurons, mitochondrial homeostasis is maintained through coordinated regulation of energy production, calcium buffering, ROS control, mitochondrial dynamics, and mitophagy (14). Once this balance is disrupted, mitochondria shift from supporting neuronal survival to amplifying cell death cascades (15). Although traumatic nerve injury, cerebral ischemia, and retinal neuropathy differ anatomically and clinically, they share several convergent mitochondrial mechanisms, including bioenergetic collapse, oxidative stress, calcium overload, MPTP opening, cytochrome c release, excessive mitochondrial fission, defective mitophagy, and sterile neuroinflammation (16, 17). The following subsections therefore compare these disorders according to their common mitochondrial injury program while retaining disease-specific pathological features.

### Mitochondrial dysfunction in traumatic nerve injury

Traumatic neurological injuries, including peripheral nerve injury, spinal cord injury, and traumatic brain injury, remain major causes of disability and chronic neurological impairment (18, 19). After nerve trauma, mitochondria are rapidly affected by mechanical disruption, calcium overload, inflammatory mediators, and oxidative stress. Injured axons require substantial ATP for ion gradient restoration, cytoskeletal remodeling, axonal transport, and regeneration. However, mitochondrial damage compromises local energy supply, thereby impairing axonal repair and functional recovery (20, 21). Peripheral nerve injury can induce altered iron metabolism, mitochondrial atrophy, mitochondrial swelling, increased membrane density, and oxidative stress (22). In spinal cord injury, secondary mitochondrial damage contributes to calcium overload, excitotoxicity, ROS production, and neuronal death (23). Mitochondria are also essential for axonal development and regeneration, influencing axon extension, branching, growth cone function, and local metabolic support (24). Therefore, traumatic nerve injury creates a pathological environment in which mitochondria simultaneously suffer structural injury and fail to meet the high energy requirements of repair. At the cell death level, ROS accumulation and calcium overload promote MPTP opening and loss of mitochondrial membrane potential. This process facilitates mitochondrial outer membrane permeabilization and cytochrome c release from mitochondria into the cytosol, activating caspase-dependent apoptosis. Excessive mitochondrial fission, particularly through dynamin-related protein 1 (Drp1), further fragments the mitochondrial network and accelerates axonal degeneration. When mitochondrial biogenesis and mitophagy are insufficient, damaged mitochondria accumulate and perpetuate oxidative damage. These mechanisms make mitochondrial preservation a key therapeutic strategy for traumatic nerve injury and neuropathic pain.

### Mitochondrial dysfunction in cerebral ischemia

Cerebral ischemia results from insufficient blood supply to brain tissue, leading to oxygen and glucose deprivation, ATP depletion, ionic imbalance, glutamate excitotoxicity, and neuronal death (25, 26). Mitochondria are among the earliest and most vulnerable targets of ischemic injury (27). Ischemia and reperfusion induce respiratory chain dysfunction, ROS bursts, calcium overload, mitochondrial swelling, loss of membrane potential, apoptotic signaling, impaired mitochondrial dynamics, and inflammatory activation (28–30). One of the central events in ischemic mitochondrial injury is MPTP opening. Accumulation of ROS and intracellular calcium can inactivate mitochondrial complex I and trigger MPTP opening, resulting in mitochondrial depolarization and release of pro-apoptotic factors such as cytochrome c (31–34). These events activate caspase cascades and contribute to delayed neuronal death.

Mitochondrial dynamics are also profoundly altered after cerebral ischemia. A balance between mitochondrial fission and fusion is required to maintain mitochondrial quality and cellular homeostasis (35). After ischemia/reperfusion, excessive Drp1-dependent mitochondrial fission is frequently observed and is associated with neuronal death, calcium dysregulation, oxidative stress, and poor neurological outcome (36–38). Conversely, mitochondrial fusion proteins such as optic atrophy 1 (Opa1) are important for preserving mitochondrial cristae structure, preventing cytochrome c release, and maintaining mitochondrial network integrity (36, 39). In addition to apoptosis, damaged mitochondria contribute to post-ischemic neuroinflammation. Mitochondrial ROS and mitochondrial DNA can act as damage-associated molecular patterns that activate the NLR family pyrin domain containing 3 (NLRP3) inflammasome (40, 41). NLRP3 activation promotes the maturation and release of IL-1β and IL-18, amplifying neuroinflammation and secondary brain injury. Mitophagy, especially through PINK1/Parkin-related pathways, can attenuate ischemic neuronal injury by selectively clearing dysfunctional mitochondria (42, 43). However, when mitophagy is insufficient or dysregulated, damaged mitochondria persist and worsen neuronal injury.

### Mitochondrial dysfunction in retinal neuropathy

Retinal neurons, especially retinal ganglion cells, have high metabolic demand and are particularly vulnerable to mitochondrial injury. Mitochondrial dysfunction is implicated in diabetic retinopathy, glaucoma, retinal ischemia, age-related macular degeneration, retinopathy of prematurity, and optic nerve injury (44, 45). Because retinal ganglion cells possess long axons and rely heavily on mitochondrial energy supply, disruption of mitochondrial homeostasis can rapidly lead to axonal degeneration and cell death. In diabetic retinopathy, hyperglycemia induces mitochondrial DNA damage, oxidative stress, mitochondrial swelling, membrane damage, and impaired respiratory function (44, 45). Retinal mitochondria from diabetic conditions show reduced levels of mitochondrial fusion proteins such as Mitofusin 2, suggesting impaired mitochondrial fusion and quality control (46). High glucose can also activate protein kinase C delta (PKCδ)/Drp1 signaling, promoting excessive mitochondrial fission and mitochondrial dysfunction (46).

In glaucoma and optic nerve injury, retinal ganglion cell death is associated with mitochondrial depolarization, ROS accumulation, cytochrome c release, and activation of caspase-dependent apoptosis. Retinal ischemia and excitotoxicity similarly cause mitochondrial fragmentation and oxidative damage. Studies of light-induced retinal degeneration have also revealed impaired mitochondrial dynamics and mitochondrial fragmentation in retinal cells exposed to long-term blue light (47). The ability of mitochondrial transplantation to protect against optic nerve injury further supports mitochondria as a therapeutic target in retinal neuropathy (48). Taken together, traumatic nerve injury, cerebral ischemia, and retinal neuropathy share a common mitochondrial injury program, despite their different initiating insults. This program includes bioenergetic failure, ROS accumulation, calcium dysregulation, MPTP opening, cytochrome c release, mitochondrial fission/fusion imbalance, defective mitophagy, apoptosis, and inflammation. These shared mechanisms provide the rationale for the next section, which examines PACAP as a candidate regulator capable of restoring mitochondrial homeostasis across distinct neurological injury contexts.

### PACAP signaling as an orchestrator of mitochondrial recovery and neuroprotection

PACAP exerts its biological actions mainly through three G protein-coupled receptors: PAC1, VPAC1, and VPAC2. Among these, PAC1 displays the highest affinity for PACAP and has been strongly implicated in neuroprotective signaling. After receptor activation, PACAP stimulates adenylate cyclase and increases intracellular cyclic adenosine monophosphate (cAMP), leading to activation of protein kinase A (PKA) and exchange protein directly activated by cAMP (Epac). These proximal pathways interact with downstream signaling modules, including extracellular signal-regulated kinase (ERK), phosphoinositide 3-kinase/Akt (PI3K/Akt), cAMP response element-binding protein (CREB), and inhibition of stress kinases such as c-Jun N-terminal kinase (JNK) and p38 (6, 49–51). In relation to the mitochondrial injury program outlined above, PACAP signaling can be viewed as a coordinating network that links receptor activation to mitochondrial bioenergetics, redox control, organelle dynamics, quality control, apoptosis, and inflammation. Rather than acting through a single linear pathway, PACAP functions as a pleiotropic regulator of mitochondrial resilience.

PACAP supports mitochondrial bioenergetics by maintaining mitochondrial membrane potential, ATP production, and cellular metabolic adaptation under stress (3). In injured neurons and axons, energy failure is a critical determinant of survival and regeneration (14). PACAP-mediated activation of cAMP/PKA, PI3K/Akt, and CREB-dependent signaling may enhance glucose metabolism, promote mitochondrial function, and sustain ATP production (52). This effect is particularly important in ischemic neurons, where oxygen and glucose deprivation cause rapid ATP depletion, and in injured axons, where energy supply is required for axonal regeneration and synaptic repair. Moreover, PACAP has been shown to reduce ROS accumulation and enhance antioxidant defenses (53). This may involve CREB-mediated transcriptional regulation of antioxidant enzymes, modulation of superoxide dismutase-related pathways, and inhibition of stress kinase signaling (53). By limiting ROS production, PACAP interrupts the vicious cycle between mitochondrial damage and oxidative stress.

In addition, mitochondrial fission and fusion are essential for maintaining mitochondrial network integrity and quality control (54). Excessive Drp1-dependent fission is observed in cerebral ischemia, diabetic retinopathy, optic nerve injury, and traumatic nerve damage (54, 55). Fragmented mitochondria are more susceptible to depolarization, ROS production, and removal failure (54). PACAP signaling may suppress pathological mitochondrial fission, potentially through PKA-mediated regulation of Drp1 activity, while supporting mitochondrial fusion through Opa1- and Mitofusin-related mechanisms (54, 56). Restoration of the fission-fusion balance helps preserve calcium buffering, axonal transport, ATP distribution, and neuronal survival. Mitophagy is also essential for the selective removal of damaged mitochondria (54). In neurological injury, defective mitophagy leads to accumulation of dysfunctional mitochondria that continue to generate ROS and inflammatory signals (3). PACAP may promote mitochondrial quality control by enhancing PINK1/Parkin-mediated mitophagy and supporting the clearance of damaged mitochondria (3, 54). This effect is especially relevant in cerebral ischemia, where damaged mitochondria can activate inflammatory pathways, and in retinal neuropathy, where impaired mitochondrial turnover contributes to progressive neuronal loss (17).

Mitochondria-dependent apoptosis is a central cell death pathway in neuronal injury (15). Calcium overload and oxidative stress induce MPTP opening and mitochondrial outer membrane permeabilization, resulting in cytochrome c release from mitochondria into the cytosol and activation of caspase cascades (53). PACAP inhibits this process through several mechanisms. It stabilizes mitochondrial membrane potential, suppresses MPTP opening, inhibits cytochrome c release, activates pro-survival pathways such as PI3K/Akt and ERK, and increases anti-apoptotic signaling involving Bcl-2 (56). In retinal ganglion cells, PACAP has been shown to attenuate optic nerve injury-induced apoptosis through activation of the CREB-Bcl-2 pathway (57).

**Figure 1:**
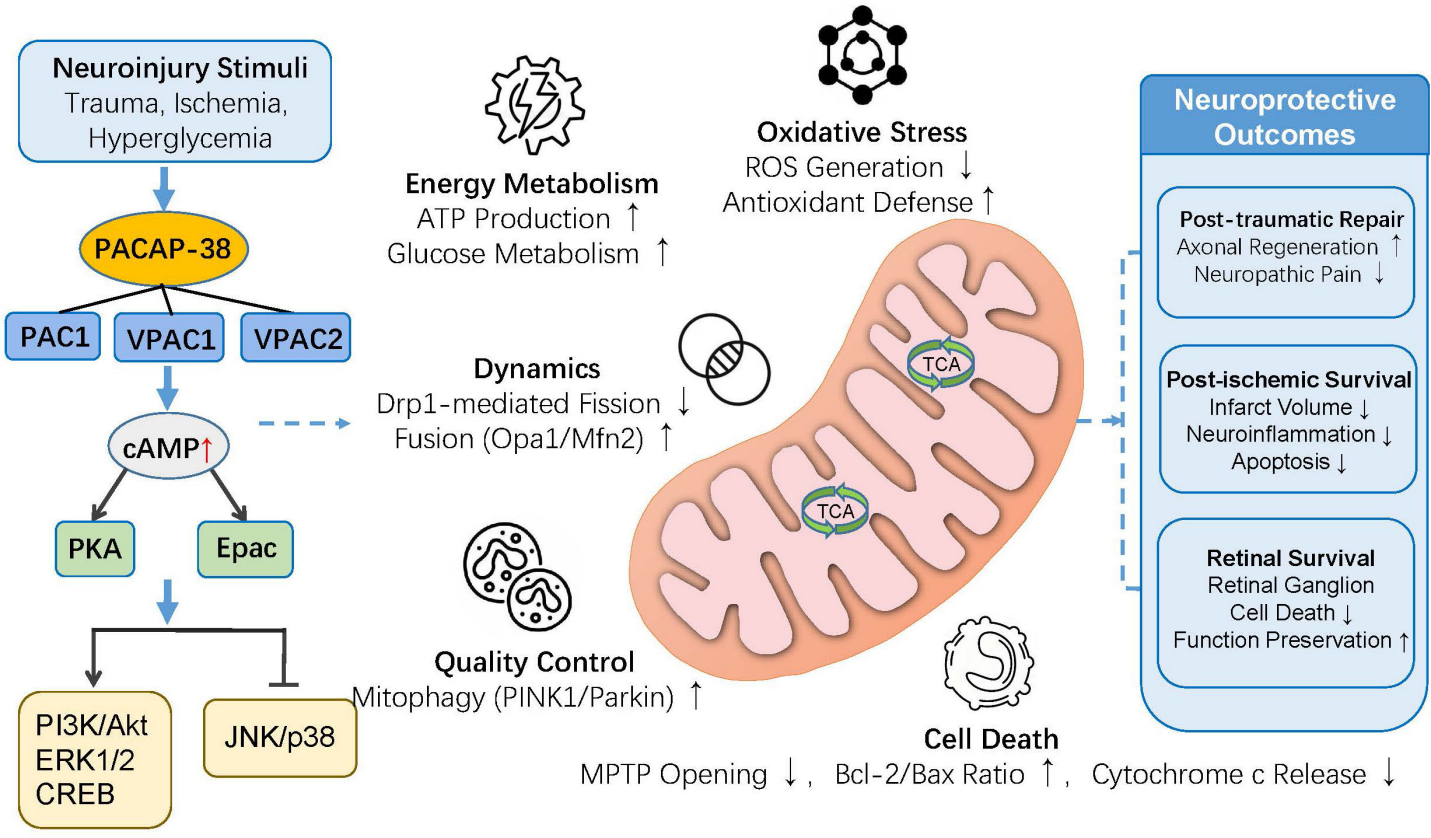
Schematic illustration of the PACAP-mitochondria axis in neuroprotection. After neurological insults such as trauma, cerebral ischemia, or retinal injury, mitochondrial dysfunction triggers MPTP opening, loss of mitochondrial membrane potential, ROS accumulation, and cytochrome c release from mitochondria into the cytosol, thereby activating downstream apoptotic cascades. PACAP-38 binds to PAC1, VPAC1, and VPAC2 receptors and activates cAMP/PKA/Epac-dependent signaling, together with PI3K/Akt, ERK, and CREB pathways, while suppressing stress-related JNK/p38 signaling. These pathways converge on mitochondria to enhance ATP production, reduce oxidative stress, rebalance mitochondrial fission and fusion, promote mitophagy, inhibit MPTP opening, block cytochrome c leakage, and suppress mitochondria-dependent apoptosis and inflammation.

Mitochondrial dysfunction also contributes to sterile inflammation (16). Mitochondrial ROS and mitochondrial DNA can activate the NLRP3 inflammasome, promoting IL-1β and IL-18 release and amplifying neuroinflammatory injury (40). By preserving mitochondrial integrity, reducing ROS generation, and promoting clearance of damaged mitochondria, PACAP can indirectly suppress NLRP3 inflammasome activation (3). In ischemic injury, this mechanism may contribute to the anti-inflammatory effects of PACAP and its ability to reduce secondary neuronal damage (52). Overall, PACAP regulates mitochondrial homeostasis through integrated effects on energy metabolism, oxidative stress, mitochondrial dynamics, mitophagy, apoptosis, and inflammation (**Figure 1**). These mechanistic links provide the basis for its protective effects across anatomically distinct neurological disorders and lead naturally to the preclinical evidence summarized below.

### Preclinical evidence supporting PACAP-mediated mitochondrial neuroprotection

A growing body of preclinical evidence supports the neuroprotective role of PACAP in traumatic nerve injury, cerebral ischemia, and retinal neuropathy (**Table 1**). Across these models, PACAP consistently modulates mitochondrial damage-associated pathways, including oxidative stress, apoptosis, mitochondrial dynamics, and inflammatory responses (54). In traumatic nerve injury, PACAP expression increases in neuronal cell bodies after nerve damage. Jongsma et al. demonstrated that unilateral sciatic nerve transection altered the distribution of PACAP and PACAP receptor binding sites in the dorsal root ganglion and spinal cord over time, suggesting a role in injury response and regeneration (58). Similar findings showed increased PACAP immunoreactivity in neuronal cell bodies after sciatic nerve compression (59). In a nerve-free crush model, single-cell RNA sequencing identified Adcyap1, the gene encoding PACAP, as a key gene linking pain and nerve regeneration. Repeated intrathecal administration of PACAP-38 reduced pain and facilitated axonal regeneration, whereas Adcyap1 siRNA or PACAP6-38 delayed axonal regeneration and increased mechanical hypersensitivity (60). These findings support PACAP as an endogenous compensatory factor and a potential therapeutic target in traumatic nerve injury.

**Table 1:** Mitochondrial dysfunction in different neurological injury models and the corresponding protective strategies of PACAP.

Injury model / pathological context	Key features of mitochondrial dysfunction	Core mitochondrial targets and mechanisms of PACAP Intervention	Key signaling pathways Involved	Primary neuroprotective outcomes
Traumatic Nerve Injury (e.g., Sciatic Nerve Crush, Spinal Cord Injury)	Bioenergetic crisis (ATP deficit in axons). Disrupted iron metabolism & increased oxidative stress. Imbalanced dynamics (shift towards fission). Calcium overload & apoptosis initiation.	Promotes bioenergetics to support axonal regeneration. Alleviates oxidative damage, maintaining redox homeostasis. May inhibit excessive Drp1-mediated fission to preserve mitochondrial networks. Exerts anti-apoptotic effects (inhibits cytochrome c release).	cAMP/PKA/CREB. PI3K/Akt. Inhibition of JNK/p38.	Enhanced axonal regeneration (25). Attenuated neuropathic pain (25, 26). Improved neuronal survival (22).
Cerebral Ischemia/Reperfusion Injury	MPTP opening and permeability transition. Severe dynamics imbalance (excessive Drp1-mediated fission). Impaired mitophagy. mtDNA/ROS release, activating the NLRP3 inflammasome.	Stabilizes the MPTP to prevent swelling and rupture. Restores dynamics balance (inhibits Drp1, promotes fusion). Enhances mitophagy to clear damaged units. Suppresses NLRP3 inflammasome activation.	PKA (e.g., phosphorylating Drp1 at Ser637). ERK. PI3K/Akt. cAMP/Epac.	Reduced cerebral infarct volume (55). Diminished brain edema (57). Attenuated neuroinflammation (58, 59). Decreased apoptosis (7, 53).
Retinal Neuropathy (e.g., Diabetic Retinopathy, Glaucoma, Light-Induced Damage)	High glucose/oxidative stress-induced dynamics disorder (increased fission). Decreased mitochondrial membrane potential in retinal ganglion cells. Activation of apoptotic pathways (altered Bcl-2/Bax ratio).	Corrects dynamics imbalance (e.g., inhibits PKCδ/Drp1 signaling). Reduces ROS production, protecting photoreceptor cells. Exerts anti-apoptotic effects (activates CREB-Bcl-2 pathway).	cAMP/PKA/CREB. MAPK (ERK). PI3K/Akt.	Increased survival of retinal ganglion cells (69, 71, 73). Improved electroretinogram function (73). Reduction in pathological neovascularization (73).

In cerebral ischemia, PACAP has shown protective effects in both *in vitro* and *in vivo* models. PACAP prevented ischemia-induced neuronal death in the hippocampal CA1 region in rat forebrain ischemia models (8). It reduced brain damage after global and focal ischemia, decreased infarct size, improved functional outcomes, and suppressed ischemic edema (61, 62). Mechanistically, PACAP has been associated with inhibition of JNK and p38 pathways, activation of ERK signaling, stimulation of astrocytic interleukin-6 release, and activation of apurinic/apyrimidinic endonuclease 1/reduction-oxidation factor 1 (APE1/Ref-1) (52, 61–66). These pathways converge on mitochondrial protection by reducing oxidative stress, apoptosis, and inflammatory injury.

In retinal neuropathy, PACAP has demonstrated protective effects in models of retinal ischemia, glutamate-induced excitotoxicity, optic nerve crush, acute ocular hypertension, oxidative retinal injury, and diabetic retinopathy (57, 67–72). PACAP and PAC1 receptor expression are upregulated in retinal ganglion cells after optic nerve crush, suggesting an endogenous protective response (69, 70). PACAP attenuates retinal ganglion cell death through cAMP, MAPK, CREB, and Bcl-2-related pathways (57, 69, 71). In diabetic retinopathy, PACAP promotes neuronal survival and reduces retinal injury by modulating apoptotic and survival signaling (72). PACAP also enhances retinal ganglion cell viability, inhibits ROS production, and increases anti-apoptotic protein expression under stress conditions (73).

### Translational barriers and future therapeutic strategies

Although PACAP has shown robust neuroprotective effects in experimental models, the transition from mechanistic promise to clinical application remains challenging. Several major barriers must be overcome before PACAP-based therapies can be translated into neurological practice, including short plasma half-life, poor blood–brain barrier penetration, receptor complexity, peripheral side effects, and insufficient clinical biomarker validation.

One of the most important obstacles to PACAP translation is its short plasma half-life. PACAP is rapidly degraded by peptidases, and its plasma half-life after systemic administration is generally estimated to be approximately 5-10 minutes (74–77). This short half-life limits sustained receptor activation and reduces therapeutic exposure in target neural tissues. Therefore, native PACAP may be unsuitable for systemic clinical application without pharmacokinetic optimization. Several strategies may be considered to overcome this limitation. First, metabolically stable PACAP analogs can be developed by modifying protease-sensitive sites (77). Second, cyclized or lipidated PACAP derivatives may increase resistance to enzymatic degradation and improve tissue penetration. Third, D-amino acid substitution, N-terminal modification, PEGylation, or fusion to carrier molecules may extend systemic circulation time. Fourth, sustained-release formulations can maintain therapeutic levels over a longer period while reducing dosing frequency. Importantly, these modifications must preserve receptor affinity and neuroprotective signaling while minimizing off-target effects.

Because PACAP is a peptide, systemic administration may result in insufficient central nervous system bioavailability. The blood-brain barrier restricts the entry of many peptide therapeutics, and high systemic doses may be required to achieve effective brain concentrations. However, high systemic exposure may increase peripheral adverse effects. Brain-targeted delivery systems are therefore essential. Intranasal administration represents a promising non-invasive route that may bypass the blood–brain barrier through olfactory and trigeminal pathways (75, 78–80). Nanoparticle-based carriers, liposomes, exosomes, cell-penetrating peptide conjugates, and receptor-mediated transport systems may further improve central delivery (80–82). For focal brain injury, local sustained-release systems may provide prolonged PACAP exposure at the lesion site while reducing systemic exposure. For example, hydrogel-nanoparticle composite systems have recently been developed for local and sustained delivery of PACAP to the post-stroke brain, producing functional improvement and reduced neuronal death in experimental models (83).

PACAP activates PAC1, VPAC1, and VPAC2 receptors, which are distributed in both neural and peripheral tissues. This broad receptor distribution contributes to PACAP’s pleiotropic biological actions but also complicates clinical translation. Systemic PACAP administration may cause vasodilation, hypotension, or migraine-like effects. Indeed, PACAP signaling is increasingly recognized as an important pathway in migraine pathophysiology (74). Clinical studies have shown that PACAP-38 infusion can induce headache attacks in migraine patients, and monoclonal antibodies targeting PACAP signaling have entered clinical trials (75). Future drug development should therefore focus on receptor subtype-selective agonists or biased agonists that preferentially activate neuroprotective intracellular pathways while minimizing vascular or nociceptive signaling. The failure of some PAC1 receptor-targeting strategies in migraine prevention suggests that PACAP receptor biology is complex and may involve different receptor subtypes, splice variants, and tissue-specific signaling mechanisms (74, 84, 85). Clarifying the role of PAC1, VPAC1, VPAC2, and PAC1 splice variants in neurons, astrocytes, microglia, endothelial cells, Schwann cells, and retinal ganglion cells will be essential for improving therapeutic specificity.

PACAP levels in plasma, serum, cerebrospinal fluid, and tissues have been proposed as potential biomarkers of neurological injury severity and prognosis. Elevated PACAP levels have been reported in several neurological conditions, including traumatic brain injury, subarachnoid hemorrhage, intracerebral hemorrhage, and neuropathic pain (86). These findings suggest that PACAP may serve not only as a therapeutic candidate but also as a prognostic biomarker. However, clinical biomarker development remains incomplete. Large-sample longitudinal studies are required to establish reference ranges, sampling windows, assay reproducibility, and correlations between PACAP levels and clinical outcomes. Standardized ELISA or mass spectrometry-based assays will be necessary to validate PACAP as both a prognostic biomarker and a pharmacodynamic marker in future therapeutic trials (87). Such assays may also help identify patient subgroups most likely to benefit from PACAP-based interventions.

## Combination therapies and future perspectives

Because mitochondrial dysfunction is multifactorial, PACAP may be most effective when combined with other mitochondrial or neuroprotective strategies. Future studies should therefore evaluate whether PACAP can complement hypothermia, mitochondrial transplantation, stem cell-based treatment, antioxidants, anti-inflammatory agents, or other neurotrophic factors. Such approaches may amplify mitochondrial protection by targeting complementary mechanisms, but they will require careful optimization of timing, dosing, delivery route, and safety profiles.

Future research should prioritize several directions that directly address the mechanistic and translational gaps identified in this review. First, cell-type-specific conditional knockout models are needed to distinguish neuronal, astrocytic, microglial, endothelial, Schwann cell, and retinal ganglion cell responses to PACAP. Second, optimized PACAP analogs with improved stability, receptor selectivity, and reduced peripheral adverse effects should be developed. Third, non-invasive brain-targeted delivery systems, especially intranasal administration and nanoparticle-based carriers, should be further evaluated. Fourth, local sustained-release systems should be optimized for stroke, traumatic injury, and retinal disease. Fifth, large-scale clinical biomarker studies should define the relationship between PACAP levels, injury severity, treatment response, and long-term neurological prognosis. Together, these strategies may help transform PACAP from a promising experimental neuroprotective peptide into a clinically viable mitochondrial-targeted therapeutic candidate.

## Conclusion

PACAP is an endogenous pleiotropic neuropeptide that protects neurons by preserving mitochondrial homeostasis. Across traumatic nerve injury, cerebral ischemia, and retinal neuropathy, PACAP supports mitochondrial bioenergetics, reduces oxidative stress, restores fission–fusion balance, promotes mitochondrial quality control, and inhibits mitochondria-dependent apoptosis and inflammation. By organizing current evidence around a shared mitochondrial injury program, this review highlights PACAP as a mechanistically coherent neuroprotective candidate rather than a disease-specific intervention. Nevertheless, clinical translation remains limited by its short plasma half-life, poor blood–brain barrier penetration, receptor complexity, and potential peripheral side effects. Future development of stable analogs, receptor-selective agonists, targeted delivery systems, and validated biomarker strategies will be essential for advancing PACAP-based neurotherapeutics.
